# Neutrophil-Related Oxidants Drive Heart and Brain Remodeling After Ischemia/Reperfusion Injury

**DOI:** 10.3389/fphys.2019.01587

**Published:** 2020-02-04

**Authors:** Federico Carbone, Aldo Bonaventura, Fabrizio Montecucco

**Affiliations:** ^1^First Clinic of Internal Medicine, Department of Internal Medicine, University of Genoa, Genoa, Italy; ^2^IRCCS Ospedale Policlinico San Martino Genoa – Italian Cardiovascular Network, Genoa, Italy; ^3^Pauley Heart Center, Division of Cardiology, Department of Internal Medicine, Virginia Commonwealth University, Richmond, VA, United States; ^4^First Clinic of Internal Medicine, Department of Internal Medicine and Centre of Excellence for Biomedical Research, University of Genoa, Genoa, Italy

**Keywords:** inflammation, reactive oxygen species, neutrophils, acute myocardial infarction, stroke

## Abstract

The inflammatory response associated with myocardial and brain ischemia/reperfusion injury (IRI) is a critical determinant of tissue necrosis, functional organ recovery, and long-term clinical outcomes. In the post-ischemic period, reactive oxygen species (ROS) are involved in tissue repair through the clearance of dead cells and cellular debris. Neutrophils play a critical role in redox signaling due to their early recruitment and the large variety of released ROS. Noteworthy, ROS generated during IRI have a relevant role in both myocardial healing and activation of neuroprotective pathways. Anatomical and functional differences contribute to the responses in the myocardial and brain tissue despite a significant gene overlap. The exaggerated activation of this signaling system can result in adverse consequences, such as cell apoptosis and extracellular matrix degradation. In light of that, blocking the ROS cascade might have a therapeutic implication for cardiomyocyte and neuronal loss after acute ischemic events. The translation of these findings from preclinical models to clinical trials has so far failed because of differences between humans and animals, difficulty of agents to penetrate into specific cellular organs, and specifically unravel oxidant and antioxidant pathways. Here, we update knowledge on ROS cascade in IRI, focusing on the role of neutrophils. We discuss evidence of ROS blockade as a therapeutic approach for myocardial infarction and ischemic stroke.

## Introduction

Neutrophil (PMN) activation is strongly implicated in the pathogenesis of cardiovascular (CV) disease (Bonaventura et al., 2019). In addition to favoring atherosclerotic plaque vulnerability and rupture (Carbone et al., 2015a, 2018b), PMNs enhance thrombosis through different mechanisms, which include generation of neutrophil extracellular traps (NETs) (Bonaventura et al., 2018), release of proteases, and direct PMN–platelet interactions (Lisman, 2018). PMNs also have a critical role in ischemia/reperfusion injury (IRI) (Carbone et al., 2013; Garcia-Culebras et al., 2018) and tissue repair (Montecucco et al., 2017) (e.g., in the myocardium and the brain). Oxidative burst characterizes PMN activation and generates several classes of reactive oxygen species (ROS). Among the different subtypes of NADPH oxidases (NOX), NOX2 is prevalent but not specific of PMNs, being commonly expressed in cardiomyocytes, endothelial cells, fibroblasts, and neurons (El-Benna et al., 2009). Components of NOX2 enzymatic complex are located either in the cytosol (p47^phox^/p67^phox^/p40^phox^ and the GTPase Rac1/Rac2) or in the plasma membrane (flavocytochrome subunits gp91^phox^ and p22^phox^). Once assembled, the glycosylated gp91^phox^ subunit undergoes a conformational change which allows catalytic activity. Activated NOX2 then generates high concentration of superoxide anion (${\text{O}}_{2}^{\cdot -}$), which exerts a prevalent local effect due to the short life span. In addition, ${\text{O}}_{2}^{\cdot -}$ dismutation may generate hydrogen peroxide (H_2_O_2_), which, in turn, reacts to produce the hydroxyl radical (^⋅^OH). The phagocyte-specific enzyme myeloperoxidase (MPO) catalyzes the formation of hypochlorous acid (HClO) and promotes the generation of chloramines, aldehydes, ^1^O_2_, ozone (O^3^), and ^⋅^OH (Prokopowicz et al., 2012). Nitric oxide synthase (NOS) is another ROS-generating enzyme active in PMNs. Through the conversion of the L-arginine to L-citrulline, NOS produces nitric oxide (NO), which may generate peroxynitrite by interacting with ${\text{O}}_{2}^{\cdot -}$ (Szabo et al., 2007). On this basis, it is not surprising that oxidative stress largely contributes to IRI. Conversely, less is known about the potential involvement in tissue repair. In the next paragraphs, we will focus on both myocardial and brain remodeling, also discussing the potential therapeutic implication of oxidative stress modulation.

## Neutrophil Oxidative Burst: Targets and Signaling

Neutrophil-derived ROS show a specific diffusion range, determined by their life span and reactivity, whereas ${\text{O}}_{2}^{\cdot -}$ has a short life span. The non-radical compound H_2_O_2_ generated by its dismutation readily diffuses across membranes. Therefore, ROS may differently oxidize DNA, RNA, protein, and lipids. Nucleic acids undergo direct oxidative processes (e.g., nitrosative deamination, oxidation, and halogenation) or alternatively generate adducts with oxidized polyunsaturated fatty acids, protein, carbohydrates, and even nucleic acids themselves (Lonkar and Dedon, 2011). Posttranslational modification of proteins may occur through a direct oxidation of amino acids or other cellular components. Endoplasmic reticulum is extremely sensitive to the redox stress, which may determine disruption of the protein folding mechanism and the production of misfolded proteins (Cao and Kaufman, 2014). Also, catabolic processes are under the control of oxidative stress, which modulates protein degradation and autophagy (Pajares et al., 2015). By targeting polyunsaturated fatty acids, ROS may also determine membrane permeability, cytosol efflux, loss of membrane protein activities, and even biomembrane disruption with loss of cell viability (Jaganjac et al., 2016).

**FIGURE 1:**
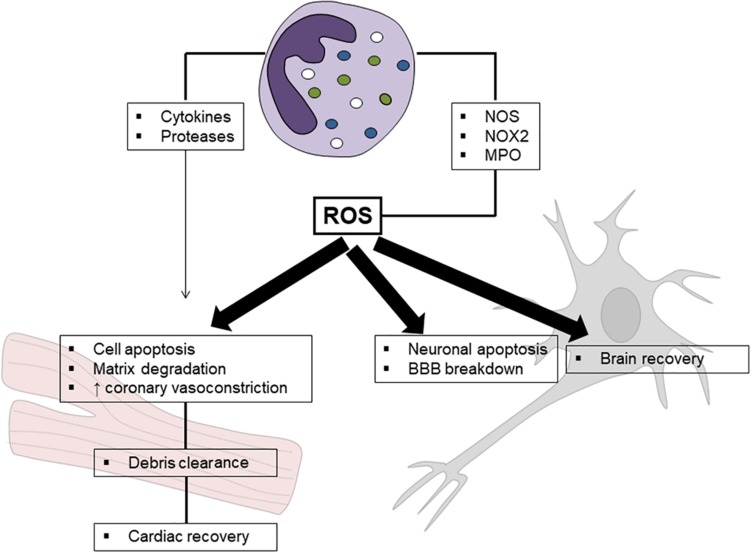
Schematic mechanism of neutrophil-related oxidative stress in ischemia/reperfusion injury. Reactive oxygen species (ROS) released by neutrophils are mainly produced by nitric oxide synthase (NOS), NADPH oxidase type 2 (NOX2), and myeloperoxidase (MPO). Although their detrimental role in ischemia reperfusion injury has been clearly established, a potential effect in promoting tissue healing has been suggested, especially in myocardial injury.

Finally, ROS may themselves act as second messengers and then transduce signals. Mitogen-activated protein kinases (MAPKs) are regulated by oxidative stress via different signaling cascades, involving Jun, p38 or extracellular signal-regulated kinase (ERK) 1/2 pathways, protein kinase C (PKC) and phosphoinositide 3-kinase (PI3K) activation (Hotamisligil and Davis, 2016). By adding an additional level of control, oxidative stress regulates a large amount of transcription factors (e.g., hypoxia-inducible factor [HIF]-1, activator protein [AP]-1, nuclear factor κ-light-chain-enhancer of activated B cells [NF-kB], and p53). It is then not surprising that ROS strongly influence in both autocrine and paracrine manner different PMN functions including phagocytosis, cytokine secretion, and apoptosis. Noteworthy, PMN-derived ROS also drive tissue response to IRI by modulating pathophysiological processes of resident cells (e.g., cardiomyocytes, endothelial and microglial cells, and neurons). The following paragraph will focus on this complex interaction between PMNs and the surrounding environment (Figure 1).

## Neutrophil Oxidants and Myocardial Remodeling

Myocardial reperfusion after an acute myocardial infarction (AMI) is recommended to save as much myocardium as possible from necrosis and dysfunction. Anyway, when the coronary flux is re-established, and the myocardium reperfused after ischemia, the hypercontracture of cardiac myocytes and their cytolysis may be paradoxically increased in response to reoxygenation. This phenomenon, known as “oxygen paradox,” forms a complementary dyad with the oxidative stress (Davies, 2016). The term “myocardial reperfusion injury” (MRI) describes myocardial injury and cardiomyocyte death usually occurring between 6 and 24 h after reperfusion of an ischemic area (Hausenloy and Yellon, 2016). Importantly, MRI is the main cause of death for stunned cardiomyocytes and ultimately accounts for more than half of the final size of myocardial ischemia. As widely reported in clinical and experimental studies, cardiomyocyte exposure to ROS causes apoptosis through different mechanisms. The leading one is likely the cytosolic and mitochondrial calcium overload that determines rapid alteration of intracellular pH. This, in turn, triggers the activation of MAPKs (Ong et al., 2015). Of interest, recent studies hypothesized oxidative stress as a promoter of myocardial fibrosis after ischemic injury, and both angiotensin 1 and the K^Ca3.1^ channel have been suggested as involved pathways (Somanna et al., 2016; Wang et al., 2017). PMNs are recruited very early to the infarcted area (nearly after 30 min), and their actions are mediated by adhesive interactions with vascular endothelial cells, such as selectins, integrins, and molecules belonging to the immunoglobulin superfamily (Bonaventura et al., 2016). Selectin-dependent adhesion of PMNs mainly involves L-selectin (CD62L), E-selectin (CD62E), and P-selectin (CD62P). They play a critical role in neutrophil rolling so that their blocking is effective in reducing PMN recruitment and infarct size (Weyrich et al., 1993; Palazzo et al., 1998). However, selectins alone do not allow transmigration until the integrins start to play. PMNs express a combination of the β-chain CD18 with the α-chains CD11a [LFA-1 (lymphocyte function-associated antigen-1)], CD11b (macrophage-1 antigen), or CD11c (p150,95). PMN adhesion occurs with the binding of CD11/CD18 to the ligand intercellular adhesion molecule (ICAM)-1 expressed on the endothelial surface (Jordan et al., 1999). Hence, PMNs change shape to motile cells, and transendothelial migration takes place (Smith, 2000). The latter occurs via paracellular route through junctions between adjacent endothelial cells and involves various molecules (PECAM-1, CD99, ICAM-2, endothelial cell-selective adhesion molecule, and members of the junctional adhesion molecule family). Once the infarcted zone is infiltrated, PMNs release ROS alongside cytokines and proteolytic enzymes, ultimately feeding a vicious circle through a positive feedback loop (Panth et al., 2016; Montecucco et al., 2017). MPO is detected within the infarcted myocardium, including the luminal thrombi on eroded plaques (Ferrante et al., 2010), and MPO-generated oxidants are likely to have a negligible impact on infarct size and impact largely on the adverse left ventricle remodeling and function (Vasilyev et al., 2005). In experimental models, the mechanism of neutrophil-dependent MRI was shown to be further dependent on CD18 integrin activation and ICAM-1 expression by injured cardiac cells (Albelda et al., 1994). *In vivo*, ROS are mainly released by adherent PMNs, thus suggesting the crucial role of the PMN ligand-specific adhesion to cardiomyocytes in MRI (Frangogiannis, 2015). The generation of ROS peaks within 2–10 min from coronary artery reperfusion and acts as a trigger for immune cell chemotaxis – particularly PMN – through complement activation and upregulation of cytokines and chemokines via the NF-κB pathway (Hensley et al., 2000). The role of ROS is of utmost importance in cardiac healing after AMI as it promotes the clearance of dead cells and cellular debris. However, exaggerated oxidative stress may result in detrimental consequences, such as cell apoptosis and degradation of the extracellular matrix. This occurs when antioxidant systems (i.e., catalase, glutathione peroxidase, and superoxide dismutase) and intracellular antioxidants are overcome and the catastrophic actions of ROS may take place (Frangogiannis, 2015).

Cytochrome P-450 (CYP), xanthine oxidase (XO), NOX, monoamine oxidase (MAO), and the mitochondrial electron transport chain (METC) are the most common enzymes involved in ROS production. CYP is likely the most known source of ROS in reperfused infarcted heart (Vinten-Johansen, 2004). CYP-derived ROS increase in an oxygen-dependent way following increased uncoupling reaction and oxygen supply (Hernandez-Resendiz et al., 2018). In particular, the release of O^–2^ and H_2_O_2_ by CYP 2C9 activates the NF-κB pathway which in turn upregulates the production of proinflammatory cytokines and the expression of adhesion molecules (Fleming et al., 2001). Similarly, the overexpression of CYP 2C8 was reported to favor ROS generation, finally exacerbating coronary vasoconstriction and increasing infarct size (Edin et al., 2011). XO derives from xanthine dehydrogenase upon myocardial reperfusion and reacts with purine substrates and O_2_ to finally generate ${\text{O}}_{2}^{-}$ and H_2_O_2_. XO is usually abundant within the vascular endothelium of normal hearts and is referred to as a primary source of ROS (Hernandez-Resendiz et al., 2018). This finding is largely based on the evidence that allopurinol, a known xanthine oxidoreductase, is effective in limiting the injury occurring during MRI and reducing the infarcted size (Pisarenko et al., 1994). Furthermore, XO was described to have a role in leukocyte recruitment and neutrophil adhesion in hypoxic conditions (Matsumura et al., 1998). Concerning NOX, the subtype NOX2 plays a central role and is typically overexpressed during MRI. Apart from the role in ROS generation, NOX can also indirectly provide damages via the activation of PKC-mediated phosphorylation of the cytosolic p47phox (Patterson et al., 1999). MAOs can catalyze oxidative deamination of several monoamines leading to a great production of ROS. Moreover, MAOs have a role in the production of H_2_O_2_ in the very early reperfusion period (Kunduzova et al., 2002). Finally, METC complexes are very important sources and targets of ROS arising during MRI, particularly complexes I and III (Hernandez-Resendiz et al., 2018). Mitochondrial complex I is formed by an active A-form and a deactivated D-form, the latter being the most abundant during MRI and producing ${\text{O}}_{2}^{-}$ and H_2_O_2_ (Gorenkova et al., 2013). Mitochondrial complex III is another fundamental source of ROS during reperfusion as demonstrated by the reduced activity in ischemic hearts compared to healthy ones. Interestingly, ROS burst from the mitochondria can induce the oxidation of cholesterol and the production of oxysterols, which induce interleukin-1β release in the vascular endothelial cells and the following expression of adhesion molecules for the recruitment of immune cells (Lemaire et al., 1998; Poli et al., 2013).

Apart from the known detrimental effects of PMNs in the ischemic area, current evidence suggests that PMNs can also display reparative functions by recruiting and activating mononuclear cells (Alard et al., 2015). Recently, neutrophil depletion by a specific monoclonal antibody has been reported to not affect infarct size, but rather favor the progressive deterioration of the post-AMI cardiac function (Horckmans et al., 2017). Accordingly, the positive influence of PMNs in this setting may be due to the polarization of macrophages toward a reparative phenotype mediated, at least partially, by neutrophil gelatinase-associated lipocalin.

In line with these findings, many antioxidants were studied, although clinical trials reported some controversial results, especially with mitochondria-targeted antioxidants (coenzyme Q10, mitoQ, and MTP-131) (Table 1; Argaud et al., 2005; Karlsson et al., 2010; Skyschally et al., 2010; Chiari et al., 2014; Hausenloy et al., 2014; Cung et al., 2015; Dare et al., 2015; Eleawa et al., 2015; Hernandez-Resendiz et al., 2015; Gibson et al., 2016; Ottani et al., 2016). Coenzyme Q10 was shown to decrease the infarcted area, the inflammatory burden, and the oxidative stress and to normalize left ventricle function following AMI (Eleawa et al., 2015). MitoQ was studied in IRI after heart transplantation to reduce ROS production showing to block graft oxidative damage and blunt the early proinflammatory response in the recipient. This effect would be dependent on reducing mitochondrial DNA damage and H_2_O_2_ (Dare et al., 2015). On the contrary, no reduction in myocardial infarct size was found in the EMBRACE STEMI trial with MTP-131 (also known as Szeto-Schiller-31 or elamipretide), a cell-permeable mitochondria-targeting peptide selectively binding to cardiolipin and optimizing mitochondrial electron transport by reducing ROS generation (Gibson et al., 2016). The EMBRACE STEMI was a multicenter, randomized, double-blind phase IIa trial evaluating the efficacy and safety of MTP-131 vs. placebo infused at a rate of 0.05 mg/kg/h for 1 h among first-time anterior STEMI subjects undergoing primary percutaneous coronary intervention for a proximal or mid left anterior descending artery occlusion. No reduction of the primary endpoint (infarct size by creatine kinase–myocardial band and area under the curve over 72 h) was reached as well as no improvement in prespecified magnetic resonance imaging, angiographic, electrocardiographic, or clinical outcomes was shown (Gibson et al., 2016). In a similar manner, cyclosporine A, a potent inhibitor of the mitochondrial permeability transition pore, showed to significantly reduce myocardial infarction size in most, but not all, experimental studies (Argaud et al., 2005; Karlsson et al., 2010; Skyschally et al., 2010). As well, in some phase II clinical trials, cyclosporine A was likely to protect the heart following an AMI (Chiari et al., 2014; Hausenloy et al., 2014). On the contrary, two large clinical trials reported disappointing results, although a clear explanation is still lacking. The CYCLE (CYCLosporinE A in Reperfused Acute Myocardial Infarction) trial of 410 patients ST elevation AMI did not find any benefit with cyclosporine A administered prior to primary percutaneous coronary intervention in terms of ST-segment resolution and enzymatic myocardial infarct size (Ottani et al., 2016). In the CIRCUS (Does Cyclosporine Improve Clinical Outcome in ST Elevation Myocardial Infarction Patients) trial conducted among 970 patients with anterior ST elevation AMI, cyclosporine A immediately before primary percutaneous coronary intervention failed to improve clinical outcomes (all-cause death, heart failure hospitalization, and adverse left ventricle remodeling) at 1 year (Cung et al., 2015). For a wider explanation on this topic, readers can be referred to the review by Hausenloy et al. (2017). Among natural molecules, curcumin was largely studied as an antioxidant molecule, with promising direct and indirect effects on ROS scavenging and myocardial remodeling. An extensive discussion on this topic can be found elsewhere (Hernandez-Resendiz et al., 2015).

**TABLE 1:** Efficacy of antioxidant compounds in myocardial ischemia/reperfusion injury.

**Author**	**Year**	**Compound**	**Study protocol**	**Correlation with stroke**
Eleawa et al., 2015	2015	CoQ10	Male Wistar rats (control, sham, MI without treatment, CoQ10 then MI)	CoQ10 pre-administration significantly reduced LV infarct area and normalized LV hemodynamic parameters. CoQ10 also decreased serum BNP and circulating inflammatory markers (TNF-α, IL-6). These effects were associated with lowered TBARS scores and concurrent increase in SOD and GSSH
Dare et al., 2015	2014	MitoQ	Heart transplant model in C57BL/6 mice [control, MitoQ; all exposed to short (30 min) or prolonged (4 h) cold preservation]	MitoQ to the donor heart protected against this I/R injury by blocking graft oxidative damage and dampening the early pro-inflammatory response in the recipient.
Gibson et al., 2016	2016	MTP-131	118 patients with anterior STEMI undergoing first-time PCI plus stenting within <4 h (i.v. MTP-131 at 0.05 mg/kg/h or appearing placebo)	MTP-131 failed in significantly reducing infarct size. MTP-131 was not associated with any improvement in magnetic resonance imaging, angiographic, electrocardiographic, or clinical outcomes
Argaud et al., 2005	2005	NIM811 (cyclosporin A derivative)	NZW rabbit [sham or I/R (10/5 min) preconditioned or not].	NIM811 increases the Ca^2+^ overload required to induce MPTP opening. NIM811 also reduced both necrotic and apoptotic cardiomyocyte death.
Skyschally et al., 2010	2010	Cyclosporin A	Göttinger minipigs [sham or I/R (90/120 min) or post-conditioning]	Both cyclosporine A at reperfusion and ischemic post-conditioning failed to reduced infarct size more than controls
Karlsson et al., 2010	2010	Cyclosporin A	Pigs [CsA (10 mg/kg) or placebo]	Cyclosporine A did not reduce IS/AAR compared with placebo. Rather, apoptosis-inducing factor protein expression was higher in the cyclosporine A group, thus suggesting a potential deleterious effect.
Hausenloy et al., 2014	2014	Cyclosporin A	78 patients undergoing elective CABG surgery [CsA (2.5 mg/kg) or placebo]	There was no significant difference in mean peak cTnT. However, in higher-risk patients peri-operative myocardial injury (post-operative cTnT) was reduced in the cyclosporine A group.
Chiari et al., 2014	2017	Cyclosporin A	61 patients undergoing elective aortic valve surgery [CsA (2.5 mg/kg) or placebo]	A significant 35% reduction of area under the curve for cTnI was observed in the cyclosporine group
Ottani et al., 2016	2016	Cyclosporin A	410 patients with anterior STEMI undergoing PCI [CsA (2.5 mg/kg) or placebo]	The two groups did not differ in TnI rise or LVEF both at day 4 and at 6 months. IS did not influence CsA efficacy.
Cung et al., 2015	2015	Cyclosporin A	970 patients with large STEMI within 6 h from onset [CsA (2.5 mg/kg) or placebo]	Cyclosporine A failed to reduce the rate of composite outcome as well as that of separate clinical components. No significant difference in the safety profile was observed between the two treatment groups.
Hernandez-Resendiz et al., 2015	2015	Curcumin	Male Wistar rats [curcumin (120 mg/kg/day) after 5/6 nephrectomy]	Curcumin restored sBP, myocardial wall thickening, LVEDV, and LVEF in nephrectomized rats. Also, it diminished MMP-2 levels and overall gelatinase activity, oxidative stress, and MPTP opening.

*AAR, area at risk; BP, blood pressure; BNP, brain natriuretic peptide; CABG, coronary artery bypass graft; CsA, cyclosporin A; cTNI, cardiac troponin I; cTNT, cardiac troponin T; I/R, ischemia/reperfusion; IL, interleukin; IS, infarct size; LV, left ventricle; LVEDV, left ventricle end diastolic volume; LVEF, left ventricle ejection fraction; MMP, metalloproteinase; MPTP, mitochondrial permeability transition pore; PCI, percutaneous coronary intervention; SOD, superoxide dismutase; STEMI, ST elevation myocardial infarction; TBARS, thiobarbituric acid reactive substances; TNF, tumor necrosis factor.*

## Neutrophil Oxidants and Brain Remodeling

Differently from other peripheral organs, the brain parenchyma does not elicit stereotypic immune responses. This is largely due to the unique anatomical composition, as endothelial, epithelial, and glial barriers tightly regulate the accessibility of immune cells. The anatomical and functional characteristics of this immune-privileged organ then imply a different response to IRI despite a significant gene overlapping (Zhang et al., 2014). The low levels of antioxidants associated with high polyunsaturated fatty acids in cellular membranes make the brain more susceptible to oxidative damage (Adibhatla and Hatcher, 2010). In the early phase of ischemic IRI, mitochondrial depolarization and activation of XO determine a first oxidative burst with ROS generation (mainly ${\text{O}}_{2}^{\cdot }$ and H_2_O_2_). They trigger PMN recruitment and activation, further precipitating IRI, even more when thrombolytic drugs are administered. Indeed, we have previously demonstrated how recombinant tissue-type plasminogen activator induces an early neutrophil degranulation via PI3K/Akt, which contributes to the increased risk of hemorrhagic transformation (Carbone et al., 2015b, c). PMNs then represent the leading source of ROS in the subacute phase of stroke. Experimental models largely emphasized the detrimental effect of NOX2 activation in the ischemic brain, being brain swelling and infarct size significantly reduced in NOX2^–/–^ mice. In this context, especially ^⋅^OH has been described as typical phagocyte ROS and amplifier of ischemic injury on neuronal cells. ^⋅^OH is generated via the Fenton reaction and has great affinity for unsaturated fatty acids, ultimately leading to peroxyl radical (ROOS) generation. In turn, ROOS trigger a cycle of lipid peroxidation that destroys cellular membranes. Products of lipid peroxidation further sustain oxidative stress by generating aldehydes, dienals, or alkanes (e.g., malondialdehyde and 4-hydroxynonenal). The consequent redox unbalance leads to neuronal apoptosis and blood–brain barrier (BBB) breakdown.

More recently, NO has been suggested as an additional mediator of IRI. At low oxygen concentrations, NO accumulates and reacts with ${\text{O}}_{2}^{\cdot }$ to generate peroxynitrite. Nitrosative stress may lead to BBB breakdown, inflammation, and caspase activation, which ultimately lead to cell apoptosis through interacting with different cellular signaling pathways including matrix metalloproteinase, high-mobility group box 1, toll-like receptors 2 and 4, poly(ADP-ribose) polymerase, Src, Rho-associated protein kinase (ROCK), and glycogen synthase kinase (GSK)-3β (Radi et al., 2015). Oxidative stress may also influence epigenetic mechanisms (i.e., DNA methylation, histone modification, microRNAs) (Zhao et al., 2016; Narne et al., 2017), well known to be implicated in neuroprotection (Felling and Song, 2015; Simon, 2016; Chandran et al., 2017). Therefore, it has been suggested that ROS may contribute themselves to neuronal recovery after ischemic stroke. In line with this hypothesis, ROS-induced activation of HIF-1α/β-catenin pathway has been associated with neuronal recovery in rats (Hu et al., 2014), whereas a biphasic role of ROS has been recently suggested also in human beings (Yang et al., 2018). Furthermore, oxidative stress (mainly H_2_O_2_ and NOX signaling) regulates neural stem and progenitor cell proliferation, self-renewal, and neurogenesis (Le Belle et al., 2011). As compared with ^⋅^OH, ROS are generated constantly as part of a normal aerobic life and are active players of several metabolic pathways ranging from cell adhesion to lipid metabolism (Roy et al., 2017). Nevertheless, the extent to which the redox status potentially contributes to brain recovery has not been established yet. Rather, a large body of evidence indicates the suppression of oxidative stress as a promising strategy to reduce brain injury. Improving reperfusion is certainly the best approach to reduce ROS generation (Carbone et al., 2018a; Chamorro, 2018; Taskiran-Sag et al., 2018). Furthermore, it is likely that antioxidant compounds tested in previous neuroprotection trials might be more effective if reperfusion therapies are co-administered (Chamorro, 2018). Among different antioxidant compounds, edaravone has already been used for years in the Far East countries. As a free radical scavenger with inhibitory effects on lipid peroxidation (Yamamoto, 2017), edaravone promotes neuroprotection when combined with thrombolysis (Logallo et al., 2011; Kono et al., 2013; Wada et al., 2014; Liu et al., 2015; Llull et al., 2015; Chamorro et al., 2017; Yamaguchi et al., 2017; Lee and Xiang, 2018; Table 2). Surprisingly, free radical scavenging properties have also been described for uric acid (UA). Serum levels of UA are classically associated with increased CV risk, but this association may rather be a compensatory mechanism (Li et al., 2015). Furthermore, when administered with recombinant tissue-type plasminogen activator, UA significantly improves the efficacy of thrombolysis (Logallo et al., 2011; Liu et al., 2015; Llull et al., 2015; Chamorro et al., 2017; Table 2). Other classes of compounds are currently under evaluation in preclinical studies and include a synthetic analog of vitamin E and specific inhibitors of NOX and NOS (Sun et al., 2018). Finally, some advances in nanomedicine are expected to improve drug delivery (Zhang et al., 2017; Shen et al., 2018) or even provide new nanoparticles with antioxidant potential (Hosoo et al., 2017).

**TABLE 2:** Efficacy of antioxidant compounds in stroke.

**Author**	**Year**	**Number of patient**	**Study design (follow-up)**	**Correlation with stroke**
**Edaravone**				
Kono et al., 2013	2013	129 stroke patients	Retrospective analysis (7 and 90 days)	Edaravone was associated with higher recanalization rate (*p* < 0.01) and better mRS (*p* < 0.01).
Wada et al., 2014	2014	6336 stroke patients	Retrospective analysis (discharge)	Edaravone improved mRS score at discharge [OR 0.74 (95% CI 0.57–0.96); *p* = 0.024], without modifying length of hospital stay, hemorrhagic transformation, or in-hospital mortality.
Yamaguchi et al., 2017	2017	8274 stroke patients from PROTECT4.5 and SITS-ISTR studies	Retrospective analysis (90 days)	The combination of edaravone with r-tPA is associated with mRS improvement in patients with NIHSS score ≥ 16 (*p* < 0.05).
Lee and Xiang, 2018	2018	38 stroke patients	Prospective randomized trial (7 and 14 days)	Edaravone group was characterized by improved NIHSS score both at days 7 and 14 (*p* < 0.05 for both). Edaravone group also showed higher recanalization and lower rate of hemorrhagic transformation and bleeding complications (*p* < 0.05 for all).
**Uric acid**				
Logallo et al., 2011	2011	1136 stroke patients	Observational (7 and 90 days)	After tertile categorization, SUA correlated with early clinical improvement (*r* = 0.012; *p* = 0.02) and long-term favorable outcome [OR 1.004 (95% CI 1.001–1.009); *p* = 0.04].
Liu et al., 2015	2015	216 stroke patients	Prospective observational (90 days)	In multivariate models, increased SUA levels were associated with excellent outcomes [OR 1.005 (95% CI 1.002–1.009); *p* = 0.033]
Llull et al., 2015	2015	411 stroke patients	Prospective interventional (90 days)	UA therapy doubled the effect of placebo in improving stroke outcome in women [OR 2.088 (95% CI 1.050–4.150); *p* = 0.036], but not in men.
Chamorro et al., 2017	2017	421 stroke patients from URICO-ICTUS trial	Prospective interventional (90 days)	The addition of UA to thrombolysis improved functional outcome [OR 6.12 (95% CI 1.08–34⋅56); *p* < 0⋅05] in absence on any safety concerns.

*CI, confidence interval; mRS, modified Rankin Scale; NIHSS, National Institute of Health Stroke Scale; OR, odds ratio; r-tPA, recombinant tissue-type plasminogen activator; RR, relative risk; SUA, serum uric acid.*

## Conclusion

Free radicals have strong oxidative properties in ischemic tissues. When reperfusion occurs, the massive generation of ROS and reactive nitrogen species leads to cell death via DNA damage, protein dysfunction, and lipid peroxidation. However, signaling pathways activated by oxidative stress are also likely to be involved in the healing processes. Appropriate consideration of the role of PMN-related oxidative stress in IRI might potentially improve current therapeutic strategies. However, several critical points should be taken into account. PMN-generated ROS may have both detrimental and beneficial roles in different models of IRI. This dual role might depend on the cell and tissue types and the timing of response (Montecucco et al., 2017). ROS activity is not limited to nearby cells/tissues. Rather there is variability in the diffusion range due to the different life span and reactivity. Finally, the side effects induced by immune suppression should be considered (Horckmans et al., 2017). Addressing these issues may explain how the results of clinical trials have so far failed to reproduce the success of preclinical studies.

## Author Contributions

All the authors wrote, made substantial corrections and contribution, and approved the final version of the manuscript to be published.

## Conflict of Interest

The authors declare that the research was conducted in the absence of any commercial or financial relationships that could be construed as a potential conflict of interest.
